# Highly efficient chitinase production from *Chitinibacter mangrovi* FCG-7^T^ and immunomodulatory efficacy of generated GlcNAc in cyclophosphamide-induced immunosuppression

**DOI:** 10.3389/fmicb.2026.1826206

**Published:** 2026-05-11

**Authors:** Longzhi Chen, Jianong Wang, Zixuan Zhang, Lixia Pan, Dengfeng Yang, Nan Li, Liyan Yang

**Affiliations:** 1 College of Life Science and Technology, Guangxi University, Nanning, Guangxi, China; 2 State Key Laboratory of Non-food Biomass Energy Technology, Guangxi Key Laboratory of Marine Natural Products and Combinatorial Biosynthesis Chemistry, Guangxi Academy of Marine Sciences, Guangxi Academy of Sciences, Nanning, Guangxi, China; 3 Guangxi Testing Center for Medical Devices, Nanning, Guangxi, China

**Keywords:** chitinase, enzymatic properties, enzyme production conditions, immunomodulation, mangrove sediments

## Abstract

Chitin, the second most abundant polysaccharide, is poorly soluble and underutilized, whereas its monomer N-acetylglucosamine (GlcNAc) has broad pharmaceutical applications. Efficient enzymatic conversion of chitin to GlcNAc remains challenging. *Chitinibacter mangrovi* FCG-7^T^, a novel chitinolytic strain isolated from mangrove sediments, exhibited exceptional chitin degradation, achieving a hydrolysis zone ratio (H/C) of 4.75 and maintained genetic stability over ten successive passages. Sequential optimization using One-Factor-at-a-Time (OFAT), Plackett-Burman designs (PBD), and Box–Behnken designs (BBD) enhanced chitinase production by 49.43-fold, yielding a final activity of 1.211 U/mL under optimized conditions (pH 7.4, 20 °C, 12 g/L powdered chitin). Purification yielded a 100 kDa bifunctional chitinase *Cm*Chi, exhibiting dual chitobiosidase and N-acetylglucosaminidase (NAGase) activities. The purified enzyme showed a specific activity of 7.96 U/mg toward colloidal chitin. *Cm*Chi demonstrated a high affinity for colloidal chitin with a *K_m_* value of 0.24 mg/mL and retained over 80% stability across a broad pH range 4.0–11.0. Furthermore, *Cm*Chi completely converted colloidal chitin to N-acetylglucosamine (GlcNAc) within 48 h and demonstrated tolerance to various reagents, including 1% methanol, acetonitrile, Tween 20, and Tween 80 (v/v). Notably, in cyclophosphamide (CTX)-induced immunosuppressed mice, GlcNAc (200 mg/kg) significantly restored body weight, thymic/splenic indices, and tissue architecture while elevating serum TNF-*α*, IL-2, IgG, and IgM (*p* < 0.05). This study establishes *Cm*Chi as an efficient biocatalyst for industrial GlcNAc production and validates the immunomodulatory potential of GlcNAc, highlighting its dual applicability in biotechnology and immunotherapy.

## 1 Introduction

Chitin, the second most abundant natural polysaccharide, consists of *β*-(1,4)-linked N-acetylglucosamine (GlcNAc) units (Beier and Bertilsson, 2013) Despite its abundance, chitin’s poor solubility in aqueous and organic solvents significantly limits its applications. In contrast, its monomeric derivative GlcNAc demonstrates excellent water solubility and biocompatibility, along with diverse pharmacological properties including anti-inflammatory, antitumor, and immunomodulatory effects (Cheng et al., 2014; Pham et al., 2016; Kubomura et al., 2017). These characteristics make GlcNAc an attractive target for pharmaceutical development, highlighting the need for efficient chitin conversion technologies to valorize chitinous biomass and enable therapeutic applications.

Traditionally, industrial GlcNAc production relies on chemical hydrolysis of chitin with strong acids/bases. While these conventional methods remain prevalent, they have notable drawbacks: suboptimal yields from uncontrolled side reactions, heterogeneous byproduct formation, and heavy environmental impacts from corrosive reagents/organic solvents (Hamed et al., 2016; Guo et al., 2017). These limitations have consequently spurred growing interest in enzymatic hydrolysis as a more sustainable alternative. Microbial chitinases offer distinct advantages over chemical approaches, including higher substrate specificity, milder reaction conditions, and enhanced environmental compatibility (Zhao et al., 2023). Nevertheless, current enzymatic processes face three major technical barriers: low enzyme production yields (Liu et al., 2019), inadequate operational stability under industrial conditions (Xie et al., 2025), and incomplete substrate conversion, leading to accumulation of mixed oligosaccharides (e.g., (GlcNAc)_2_ and (GlcNAc)_3_) rather than the desired monomeric GlcNAc (Zhou et al., 2019). For instance, although *Pseudomonas* sp. GWSMS-1 exhibits remarkable environmental adaptability, its low extracellular enzyme activity limits its potential for practical applications (Liu et al., 2019). Similarly, *Bacillus paralicheniformis* GXMU-J23.1 shows promising activity but presents challenges in enzyme purification and product homogeneity (Xie et al., 2025). These limitations emphasize the need for discovering novel chitinolytic strains capable of producing robust and highly efficient enzymes with well-defined product profiles.

Mangrove ecosystems have gained increasing attention as promising sources of extremophilic microorganisms possessing unique biocatalytic capabilities (Ghizelini et al., 2012). The challenging and dynamic mangrove environment-characterized by fluctuating salinity, anaerobic sediments, and intense nutrient competition-favors the evolution of microbes that produce highly stable enzymes (Rishad et al., 2017). Notably, chitinolytic bacteria derived from mangroves often exhibit superior environmental tolerance and catalytic efficiency compared to terrestrial strains (Rishad et al., 2016). Despite this potential, few mangrove-associated chitinases have been systematically characterized for industrial applications, indicating a significant untapped resource for bioprocess development.

Concurrently, GlcNAc has garnered attention for its immunomodulatory properties. Early work by Cheng et al. (2014) demonstrated that GlcNAc supplementation enhances innate immunity in tilapia, conferring improved survival against *Streptococcus iniae* infection. More recently, Xu et al. (2023) revealed that GlcNAc administration activates the NRF2/HO-1 signaling pathway, reduces oxidative stress, and modulates macrophage polarization, thereby effectively mitigating acute pancreatitis in rats. However, despite its promising therapeutic effects in infection and inflammation models, the immunoregulatory potential of GlcNAc remains unexplored in the context of chemotherapy-induced immunosuppression. Cyclophosphamide (CTX), a widely used alkylating chemotherapeutic agent, induces profound immunosuppression through multiple mechanisms, including neutropenia, leukopenia, lymphocytopenia, and cytokine suppression (Emadi et al., 2009; Patra et al., 2012; Zheng et al., 2017; Li et al., 2018). This well-characterized immunosuppressive effect has established CTX-treated mice as a standard model for evaluating immunomodulatory compounds (Xu et al., 2025). The current study aims to address this critical gap by employing the CTX-induced immunosuppression model to systematically investigate the immunoregulatory properties of GlcNAc. This approach will not only expand our understanding of GlcNAc’s therapeutic potential but also provide mechanistic insights into its action under clinically relevant immunosuppressive conditions.

Here, we characterize a novel chitinase from the mangrove-derived bacterium *Chitinibacter mangrovi* FCG-7^T^, addressing challenges in both biotechnological production and biomedical application. Through multistep optimization, we achieved unprecedented enzyme yields while demonstrating complete chitin-to-GlcNAc conversion. Notably, we provide the first evidence of GlcNAc-mediated immune restoration in cyclophosphamide-treated mice, thereby bridging the gap between enzymatic production and therapeutic application. This work establishes an integrated platform that links sustainable chitin valorization with immunomodulation research.

## 2 Materials and methods

### 2.1 Materials

Astragalus polysaccharide (purity >98%) was purchased from Shanghai Aladdin Biochemical Technology Co., Ltd. CTX was obtained from TCI (Shanghai) Development Co., Ltd. Enzyme-linked immunosorbent assay (ELISA) kits for tumor necrosis factor-*α* (TNF-α), interleukin-2 (IL-2), immunoglobulin G (IgG), and immunoglobulin M (IgM) were sourced from Shanghai Enzyme-linked Biotechnology Co., Ltd. Colloidal chitin was prepared from chitin powder under the previous method (Deng et al., 2019), with appropriate modifications. All other chemical reagents used are of analytical grade and readily available commercially.

### 2.2 Evaluation of chitin degradation ability and *C. mangrovi* FCG-7^T^ subculturing ability

The chitinolytic strain *C. mangrovi* FCG-7^T^ was employed as the first-generation strain (Xu et al., 2025). This strain was successively transferred onto fresh colloidal chitin agar plates to generate subsequent generations. The screening medium, referred to as colloidal chitin agar plates, contained the following components per liter: 10 g colloidal chitin, 20 g agar, 0.6 g K_2_HPO_4_, 0.4 g KH_2_PO_4_, 0.5 g MgSO_4_, and 0.02 g FeSO_4_, with the pH adjusted to 7.2–7.4. For serial passage, a single colony from each generation was aseptically transferred to fresh medium to establish the next generation. All cultures were incubated upside down at 30 °C. The colony morphology and diameter ratios were recorded after 6 days of incubation for each generation.

### 2.3 Fermentation and chitinase production

A single colony was transferred to R2A nutrient broth and incubated at 30 °C with shaking at 200 rpm for 24 h to prepare the seed culture. Subsequently, 3% (v/v) of the seed culture was inoculated into a 500-mL Erlenmeyer flask containing 100 mL of fermentation medium. Fermentation was carried out at 30 °C with orbital shaking at 200 rpm. The fermentation medium composition (g/L) was modified from Wang et al. (2023) and consisted of: colloidal chitin, 2.5; yeast extract, 2.5; peptone, 2.5; K₂HPO₄, 0.7; KH_2_PO_4_, 0.3; NaCl, 1.0; MgSO_4_, 0.5; CaCl_2_, 0.1; FeSO_4_, 0.1.

Chitinase activity was estimated using colloidal chitin as substrate. The assay mixture contained 400 μL of 1% (w/v) colloidal chitin dissolved in 0.1 M sodium phosphate buffer (pH 6.0) and 100 μL of appropriately diluted fermentation broth (enzyme source) in a total reaction volume of 500 μL. The mixture was incubated at 40 °C for 1 h with constant shaking, followed by centrifugation at 10,000 rpm for 5 min. The supernatant was further used for analysis of reducing sugars by the 3, 5-dinitrosalicylic (DNS) method (Miller, 1959). One unit (U) of chitinase activity was defined as the amount of enzyme required to release 1 μmol of GlcNAc per minute under the standard assay conditions. Protein concentration was measured by standard Bradford assay.

### 2.4 Optimization of chitinase production

#### 2.4.1 One-factor-at-a-time optimization

To obtain maximum extracellular chitinase secreted by strain *C. mangrovi* FCG-7^T^, the one-factor-at-a-time method was adopted to optimize the medium composition including carbon and nitrogen sources, carbon and nitrogen concentration, as well as the culture conditions including fermentation time, temperature, pH, inoculum volume and shaking speed (Table S1). All experiments were performed in triplicate.

#### 2.4.2 Plackett-Burman design

The magnitude of the effect of seven variables, including four dummy variables, on chitinase production by *C. mangrovi* FCG-7^T^ was evaluated by Plackett-Burman design (PBD). The variables were studied at two levels (−1 and +1; see Table S2), and the assessment was conducted through twelve experiments (N = k + 1; where k represents the number of variables). The PBD experimental design matrix, which includes all nutritional variables alongside three dummy variables, is presented in Table S2. All experiments were performed in triplicate, and the analysis was conducted using the statistical software package “Design-Expert 8.0.6” (State-Ease Inc., Minneapolis, USA).

#### 2.4.3 RSM optimization of the selected factors using the BBD

Based on the results of PBD, factors that had a great influence on enzyme activity were optimized by BBD. Three variables and three levels were used to optimize chitinase activity. Three levels of each factor were labeled as −1, 0, and +1 shown in Table S3, and enzyme activity was taken as the response value (Table S3). All experiments were performed in triplicate, and results were analyzed by Design Expert software (version 8.0.6).

### 2.5 Chitinase purification

*C. mangrovi* FCG-7^T^ was cultured in optimized medium. Following four days of incubation under shaking conditions, bacterial cells were separated from the fermentation broth by centrifugation at 8,000 rpm. Solid ammonium sulfate was added to the resulting supernatant to achieve 10–50% saturation, and the mixture was incubated overnight at 4 °C to precipitate proteins. The protein precipitate was collected by centrifugation at 12,000 rpm for 30 min at the same temperature. The pellet was resuspended in 50 mM Tris–HCl buffer (pH 8.5) and dialyzed against the same buffer overnight at 4 °C. Immediately after dialysis, chitinase activity was reassessed and the protein concentration was measured using the standard Bradford assay. The crude protein extract was loaded onto a DEAE-52 cellulose chromatography column for purification. The molecular weight of the purified chitinase was determined using 12% sodium dodecyl sulfate-polyacrylamide gel electrophoresis (SDS-PAGE). Zymogram analysis of the purified chitinase was subsequently performed according to the method described by Singh and Pachaiappan (2025) with slight modifications. Briefly, 0.1% ethylene glycol chitin was incorporated into the separation gel prior to SDS-PAGE electrophoresis. After electrophoresis, the gel was washed in phosphate buffer containing 1% triton X-100, and then stained with 0.01% Calcofluor White M2R for 5 min, followed by three washes with deionized water. Observe the band under ultraviolet light.

### 2.6 Biochemical characterization of *Cm*Chi

All biochemical characterization assays were performed in triplicate using the purified *Cm*Chi. The standard reaction mixture (total volume 500 μL) contained 400 μL of 1% (w/v) colloidal chitin in the appropriate buffer and 100 μL of purified *Cm*Chi (0.1 mg/mL), unless otherwise specified.

Optimum pH and pH stability: The optimal pH of recombinant chitinase was assessed using 100 mM citric acid-disodium hydrogen phosphate buffer (pH 3.0–8.0) and 100 mM glycine-NaOH buffer (pH 8.0–12.0) at 50 °C for 10 min. For pH stability, the enzyme was incubated in the same buffers at 4 °C for 180 min, and residual activity was measured under standard conditions (50 °C, pH 6.0, 10 min). The group showing the highest activity was set as 100%.

Optimum temperature and thermostability: The optimum temperature was determined by incubating the reaction system at 5–70 °C for 10 min in 100 mM citric acid-disodium hydrogen phosphate buffer (pH 6.0). Thermal stability was evaluated by pre-incubating the enzyme at 0–60 °C for 30 min in the same buffer, and the residual activity was then measured under standard conditions. Again, the maximum activity was taken as 100%.

Effects of metal ions and organic solvents: The effects of metal ions were assessed by adding each component (Fe^3+^, Co^2+^, Ca^2+^, Zn^2+^, Li^+^, Cu^2+^, Mg^2+^, Mn^2+^, Ni^2+^, Ba^2+^, K^+^, Na^+^, NH_4_^+^) to the standard reaction mixture at a final concentration of 10 mM. EDTA was similarly tested at a final concentration of 10 mM. The effects of organic solvents were evaluated by adding glycerol, methanol, ethanol, acetonitrile, Tween 20, or Tween 80 to the reaction mixture at a final concentration of 1% (v/v). Activity measured in the absence of any additive was defined as 100%.

Substrate specificity and kinetic parameters: Substrate specificity was determined using 0.02 g/mL of various substrates (colloidal chitin, *α*-chitin, *β*-chitin, chitosan, microcrystalline cellulose (MCC), and sodium carboxymethyl cellulose (CMC-Na)) under standard conditions. Kinetic parameters toward colloidal chitin (0.001–0.05 g/mL) were determined in 100 mM citric acid-disodium hydrogen phosphate buffer (pH 6.0) at 50 °C for 10 min. The maximal reaction rate (*V*_max_), michaelis constant (*K*_m_), turnover number (*k_cat_*), and the catalytic efficiency (*k_cat_*/*K*_m_) were calculated using GraphPad Prism 9.

Hydrolysates of colloidal chitin by *Cm*Chi: Hydrolysis products of chitinase were analyzed by High-Performance Liquid Chromatography (HPLC, UltiMate 3,000, Dionex) and Liquid chromatography-triple quadrupole mass spectrometer (LC–MS/MS, Agilent, United States). Colloidal chitin (2%) was degraded by treating it with 2 mg/mL chitinase at 40 °C for different times (1, 3, 6, and 12 h), and the reaction was terminated after boiling for 10 min. Then the produced reducing sugar was obtained by collecting the supernatant after centrifugation. The samples were filtered using a 0.22 μm filter (JET, Guangzhou, China) and analyzed through HPLC. The reaction mixture (10 μL) was injected into the HPLC system. The products were analyzed on the Phenomenex Luna NH_2_ column (250 mm × 4.6 mm) by using acetonitrile: water = 7:3 as the mobile phase at a flow rate of 0.6 mL/min at 45 °C. Chitin oligomers with different polymer degrees (DP) were used as the standard. The solvents used for LC–MS/MS were as follows: solvent A was a solution of 0.1% formic acid in H_2_O, and solvent B was acetonitrile. The LC–MS/MS system operated at a flow rate of 0.2 mL/min. Mass spectra were acquired in negative ionization mode.

NAGase activity assay: The N-acetylglucosaminidase (NAGase) activity was assayed with 4-MU-NAG as substrate according to the previous method with some modification (Litzinger et al., 2010). The reaction (5 μL enzyme + 95 μL 4-MU-GlcNAc, 50 °C, 10 min) was stopped with 100 μL glycine-NaOH (1 M, pH 10) and the released 4-MU quantified fluorimetrically (λex 360 nm, λem 460 nm). One unit enzyme activity was defined as the amount of enzyme required to produce 1 μM of free 4-MU per minute at standard conditions.

### 2.7 Preparation of GlcNAc

Purification of GlcNAc from *Cm*Chi enzymatic hydrolysates was initiated by desalting the purified enzyme, followed by a 48-h hydrolysis reaction at 30 °C using 2% colloidal chitin in ultrapure water as substrate. The reaction was terminated by boiling-water bath treatment, with subsequent centrifugation to remove unreacted substrate and denatured enzymes. GlcNAc powder was subsequently obtained through vacuum drying of the processed supernatant.

### 2.8 *In vivo* immunomodulatory activity assessment

#### 2.8.1 Experimental animals and treatments

Male inbred Balb/c mice (18–20 g; purchased from Henan Skobes Biotechnology Co., Ltd., License No. SCXK 2020–0005) were acclimatized for ≥1 week in specific pathogen-free (SPF) conditions (25 ± 2 °C, 50–55% humidity, 12-h light/dark cycle) with ad libitum access to sterilized water and standard chow. All animal procedures were approved by Guangxi University’s Ethics Committee with strict adherence to pain minimization protocols. Immunosuppression was induced via CTX administration, with animals randomized into six groups (n = 10/group): (1) Normal control (CK), (2) CTX model (CTX), (3) Positive control (APS), (4) High-dose GlcNAc (GlcNAc-H), (5) Medium-dose GlcNAc (GlcNAc-M), and (6) Low-dose GlcNAc (GlcNAc-L). Groups 2–6 received intraperitoneal CTX injections (80 mg/kg/day) on days 7–10 (Wang et al., 2012; Monmai et al., 2017; Alharbi et al., 2025), while CK received equivalent volumes of sterile saline. From day 1, APS (100 mg/kg) was administered to the positive control, with GlcNAc groups receiving oral gavage at 200, 100, and 50 mg/kg respectively; all interventions continued for 10 consecutive days (Supplementary Figure 1). All experimental procedures involving animals were approved by the Animal Ethics Committee of Guangxi University (Approval No. Gxu-2025-224) and performed in accordance with relevant guidelines.

#### 2.8.2 Body weight and immune organ index analysis

Body weights were monitored and recorded daily, while thymus and spleen tissues were collected at day 11 which were thoroughly rinsed three times with PBS, blotted dry on filter paper, and weighed. The organ index was calculated as: Organ Index = Organ Mass (g) / Final Body Weight (g).

#### 2.8.3 Histopathological analysis of thymus and spleen

Thymus and spleen tissues were collected from each experimental group, with three samples randomly selected from each group. The samples were fixed in 4% paraformaldehyde for 24 h prior to paraffin embedding, followed by hematoxylin–eosin (H&E) staining conducted by Servicebio Biotechnology Co. Subsequent histopathological analysis was conducted through light microscopic examination.

#### 2.8.4 Quantification of serum TNF-*α*, IL-2, IgG, and IgM levels

Blood samples were obtained via retro-orbital plexus bleeding and allowed to clot at room temperature for 4 h. Following centrifugation at 3,500 rpm for 10 min, supernatants were aliquoted as serum and stored at −80 °C. Concentrations of TNF-α, IL-2, IgG, and IgM were determined using commercial ELISA kits according to manufacturer’s protocols, with absorbance measured at 450 nm using a microplate reader. Biomarker concentrations were calculated against kit-provided standard curves.

### 2.9 Statistical analysis

All experiments were performed in triplicate, and data are presented as the mean ± standard error (SE). Significant differences between means were determined by Student’s t test. *p* < 0.05 was considered significant.

## 3 Results

### 3.1 Characterization of a novel chitin-degrading bacterium and its stability

**Figure 1:**
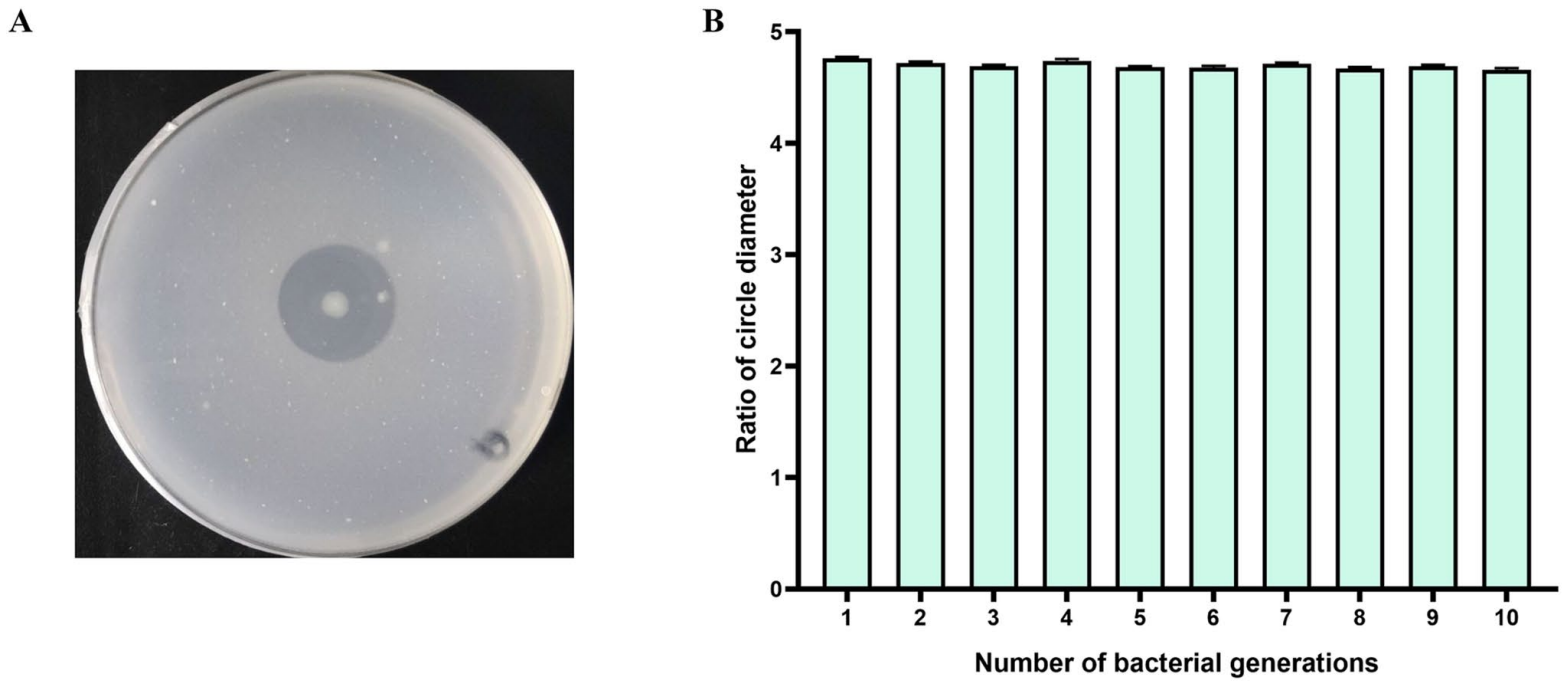
Chitinolytic activity and genetic stability of *C. mangrovi* FCG-7^T^. **(A)** Hydrolyzed zone produced by incubation of *C. mangrovi* FCG-7^T^ at 30 °C for 6 days. **(B)** Hydrolysis zone-to-colony diameter ratio (H/C) over ten consecutive subcultures. Data are presented as mean ± SE from three independent replicates (*n* = 3).

The chitinolytic strain *C. mangrovi* FCG-7^T^, previously isolated from mangrove sediments (Xu et al., 2025), was first assessed for its chitin degradation capacity. When cultured on colloidal chitin agar at 30 °C, the strain formed a clear and prominent hydrolysis zone within 6 days (Figure 1A). Quantitative analysis yielded a hydrolysis zone-to-colony (H/C) ratio of 4.75. This result confirms the strong chitinolytic potential of *C. mangrovi* FCG-7^T^ and underscores its comparative advantage over other known strains. Furthermore, the strain consistent chitin degradation activity over ten successive subcultures, as evidenced by the stable formation of hydrolysis zones (Figure 1B). This observed stability suggests that *C. mangrovi* FCG-7^T^ possesses a stable genotype and consistent enzyme production, attributes that are crucial for its potential industrial application in chitin bioconversion.

### 3.2 Optimization of chitinase production

**Figure 2:**
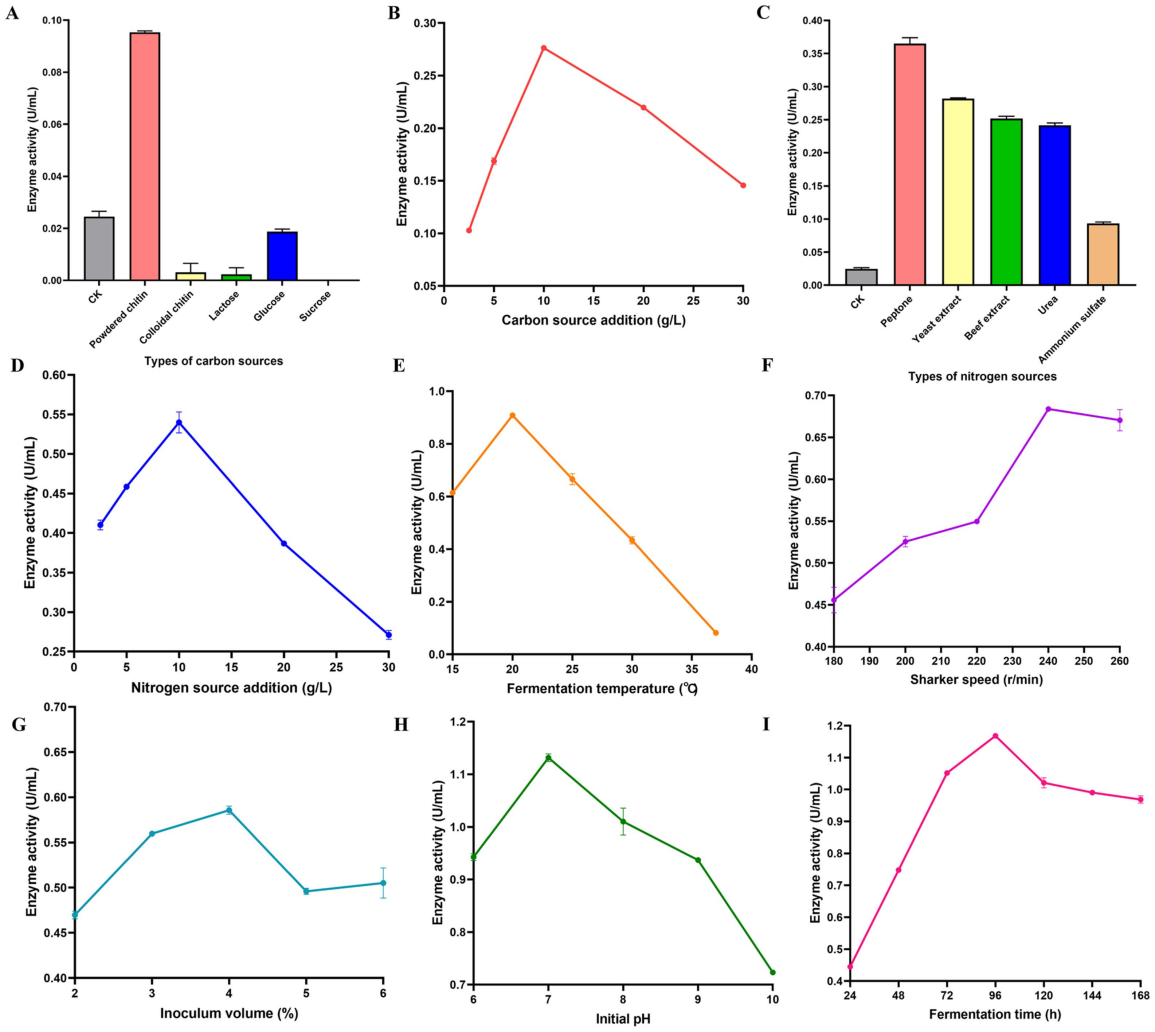
Optimization of chitinase production by *C. mangrovi* FCG-7^T^ using the one-factor-at-a-time method. Effects of **(A)** different carbon sources (each at 10 g/L) and **(B)** different concentrations of powdered chitin; **(C)** different nitrogen sources (each at 10 g/L) and **(D)** different concentrations of peptone; and fermentation conditions including **(E)** incubation temperature, **(F)** shaking speed, **(G)** inoculum volume, **(H)** initial pH, and **(I)** fermentation time. CK refers to initial fermentation medium. Data are presented as mean ± SE from three independent replicates (*n* = 3).

Carbon source selection significantly influenced chitinolytic activity. Activity was undetectable with fructose, whereas powdered chitin at 10 g/L yielded the highest activity (Figures 2A,B). Among the nitrogen sources tested, organic sources supported higher chitinolytic activity than inorganic sources (Figure 2C). Specifically, peptone at a concentration of 10 g/L conferred the highest activity (Figure 2D). Furthermore, optimal fermentation conditions were determined as follows: incubation temperature, 20 °C; shaking speed, 240 rpm; inoculation volume, 4% (v/v); initial pH, 7.0; and fermentation time, 96 h (Figures 2E–I).

Following One-Factor-at-a-Time (OFAT) optimization, a 12-run Plackett-Burman design (PDB) (7 factors, 2 levels) was used to screen chitinase production by *C. mangrovi* FCG-7^T^. Regression gave the model Y = 0.352 + 0.064A + 0.059C + 0.176F (R^2^ = 0.9692). Variables with *p* < 0.05-initial pH (F, 73.1%), chitin powder (A, 9.7%), and temperature (C, 8.3%)-were selected for further Box–Behnken optimization (Supplementary Figure 2).

Following OFAT and PBD, a 17-run Box–Behnken design (3 factors × 3 levels) was built with centre points at 10 g L^−1^ chitin, 20 °C and pH 7.0 (Table S6). The quadratic model Y = 1.08 + 0.092×_1_ + 0.045×_2_ + 0.15×_3_–0.13X_1_X_2_–0.14×_1_^2^–0.23×_2_^2^–0.20×_3_^2^ was highly significant (*p* < 0.0001) and lacked-of-fit was not (*p* = 0.197). Steep 3-D surfaces and elliptical contours revealed strong interactions, especially between chitin and temperature. Numerical optimization predicted pH 7.43, 19.9 °C and 12.1 g/L chitin; these were rounded to pH 7.4, 20 °C and 12 g/L for validation. Fermentation was conducted under optimized conditions. After 96 h of fermentation, the culture supernatants were collected and assessed for chitinase activity. The enzyme activity attained was 1.211 U/mL, representing a 49.43-fold increase compared to the pre-optimization level. These experimental results closely aligned with the model predictions, demonstrating that the model accurately reflects the influence of the studied factors on chitinase production and provides valuable guidance for optimizing chitinase manufacturing processes.

### 3.3 Purification of *Cm*Chi

**Figure 3:**
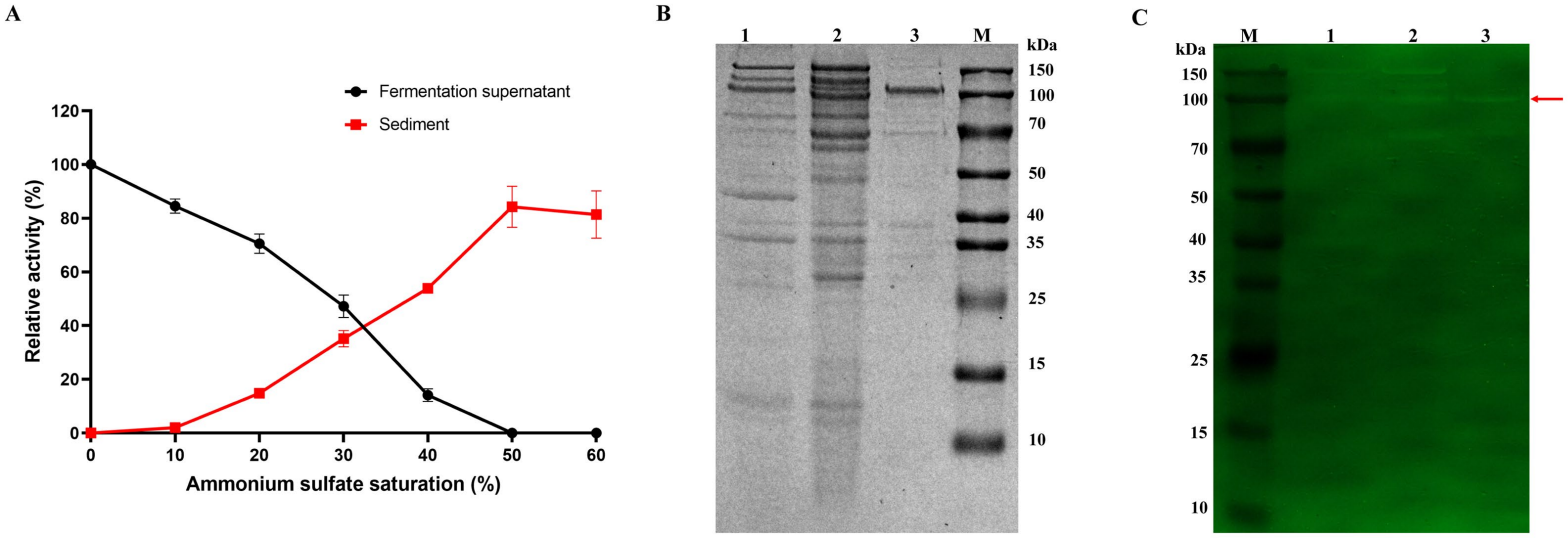
Purification and electrophoretic analysis of *Cm*Chi. **(A)** Changes in enzymatic activity at varying (NH_4_)_2_SO_4_ saturation levels. Data are presented as mean ± SE from three independent replicates (*n* = 3). 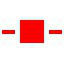, Enzyme activity of fermentation supernatant; 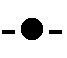, enzyme activity of sediment. M, Protein molecular weight markers. **(B)** SDS-PAGE analysis of *Cm*Chi. **(C)** Zymogram analysis of *Cm*Chi. Lane 1, crude extract; Lane 2, the resuspended ammonium sulfate precipitation fraction; Lane 3, Purified *Cm*Chi. The red arrow indicates the major chitinase band corresponding to *Cm*Chi (~100 kDa).

*Cm*Chi was enriched from 100 mL crude extract by a two-step protocol. During ammonium-sulfate fractionation the enzyme remained soluble at 10% saturation (84% activity in supernatant) and was progressively salted out as the concentration rose; precipitation was complete at 50–60% saturation, giving a 5.18-fold increase in specific activity (0.57 U/mg) (Figure 3A). The 10–50% cut was taken forward to DEAE-52 chromatography.

DEAE-52 anion-exchange chromatography showed six protein peaks (Supplementary Figure 4), only the fractions corresponding to peak I displayed chitinase activity. The specific activity of the purified target protein solution reached 7.96 U/mg, representing a 72.36-fold increase in purification compared to the initial crude enzyme preparation (Table S8). After sequential (NH_4_)_2_SO_4_ precipitation and DEAE-52 chromatography, SDS-PAGE displayed a major band of approximately 100 kDa whose chitinolytic activity was verified by zymography (Figures 3B,C), establishing *Cm*Chi as the principal chitinase of *C. mangrovi* FCG-7^T^.

### 3.4 Effect of temperature on the activity and stability of *Cm*Chi

**Figure 4:**
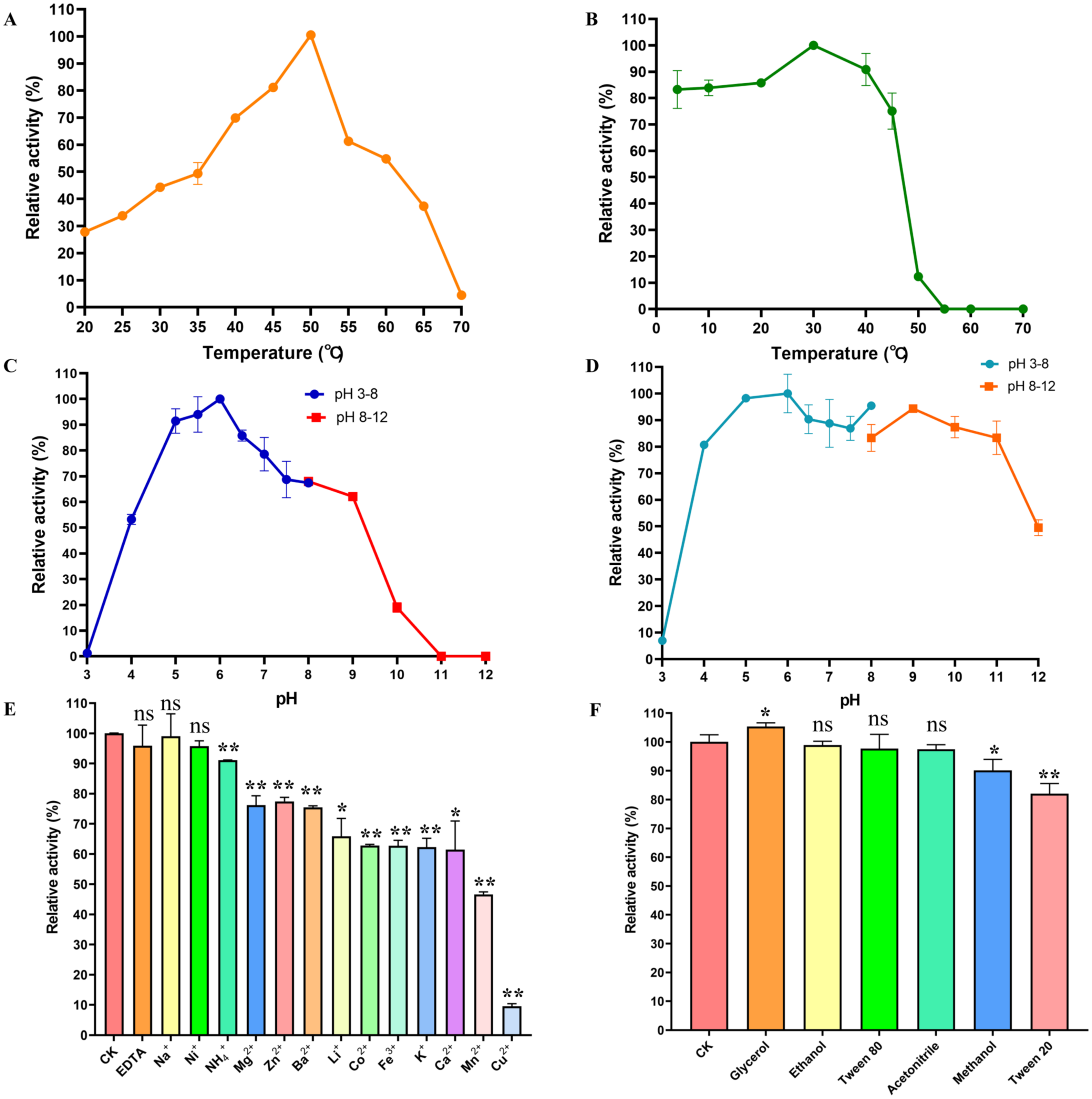
Biochemical characterization of *Cm*Chi. **(A)** The optimal temperature of *Cm*Chi. **(B)** The thermostability of *Cm*Chi. **(C)** The optimal pH of *Cm*Chi. **(D)** The pH stability of *Cm*Chi. **(E)** Effect of different ions on the enzyme activity of *Cm*Chi. The activity measured without any metal ion supplement was defined as 100% (control). Data are presented as mean ± SE (*n* = 3). Statistical analysis was performed using Student’s *t*-test. Asterisks indicate statistically significant differences compared to the control group: *p < 0.05, **p < 0.01; ‘ns’ denotes not significant (*p* > 0.05). **(F)** Effect of various organic solvents on the enzyme activity of *Cm*Chi. Data are presented as mean ± SE from three independent replicates (*n* = 3).

As demonstrated in Figure 4A, *Cm*Chi displayed a distinct temperature-activity profile, with maximum catalytic activity observed at 50 °C. Enzyme activity increased progressively with temperature from 20 °C to 50 °C, followed by a sharp decline above 60 °C, culminating in complete inactivation at 70 °C. Notably, the enzyme retained above 54.82% of its peak activity within the 50–60 °C range, indicating moderate thermotolerance. As shown in Figure 4B, *Cm*Chi displays favorable thermostability, maintaining over 70% of its initial activity after 1 h incubation at temperatures ranging from 0 °C to 45 °C.

### 3.5 Effect of pH on the activity and stability of *Cm*Chi

*Cm*Chi displayed a distinct pH-activity profile with maximum catalytic efficiency observed at pH 6.0 (Figure 4C). Enzyme activity increased progressively from pH 3.0 to 6.0, followed by gradual decline while maintaining >62.09% relative activity through pH 9.0. Complete inactivation was observed at pH 11.0, establishing the enzyme’s functional pH range. Notably, *Cm*Chi demonstrated exceptional pH stability, maintaining over 80% of its initial activity after 3 h of incubation across a broad pH range (4.0–11.0) (Figure 4D).

### 3.6 Effects of metal ions and organic solvents on *Cm*Chi activity

The effects of various metal ions at a final concentration of 10 mM on *Cm*Chi activity were systematically evaluated. As shown in Figure 4E, supplementation with EDTA, Ni^2+^or Na^+^ exerted no statistically significant effect on enzymatic activity compared to the untreated control (*p* > 0.05). In contrast, Ca^2+^ and Li^+^ caused significant but relatively modest inhibition (*p* < 0.05). More pronounced inhibitory effects were observed with NH_4_^+^, Co^2+^, Zn^2+^, Cu^2+^, Mg^2+^, K^+^, Fe^3+^, Ba^2+^, and Cu^2+^, all of which significantly reduced *Cm*Chi activity (*p* < 0.01). Notably, Cu^2+^ exhibited the most potent inhibitory effect, drastically reducing residual activity to merely 9.53% of the control. As shown in Figure 4F, at a low concentration of 1% (v/v), ethanol, acetonitrile, and Tween 80 had negligible effects on the activity of *Cm*Chi (*p* > 0.05). Methanol and Tween 20 significantly reduced *Cm*Chi activity (*p* < 0.05). Moreover, the addition of glycerol at 1% (v/v) was found to significantly enhance the hydrolytic activity of *Cm*Chi (*p* < 0.05).

### 3.7 Substrate specificity and kinetic parameters of *Cm*Chi

The activity of *Cm*Chi toward various substrates is presented in Table 1. *Cm*Chi exhibited significant activity toward colloidal chitin, *α*-chitin, *β*-chitin, and MCC, while only weak activity was detected with chitosan and CMC-Na. Among the effective substrates, *Cm*Chi displayed the highest activity toward colloidal chitin, followed by β-chitin and α-chitin.

**Table 1:** The substrate specificity of *Cm*Chi.

Substrate	Enzyme activity (U/mg)
	*Cm*Chi
Colloidal chitin	7.96
α-chitin	0.685
β-chitin	1.92
Chitosan	0.034
MCC	0.644
CMC-Na	0.017

The kinetic parameters of *Cm*Chi were determined using colloidal chitin as the substrate, and fitting calculations performed with GraphPad software yielded a *K*_m_ value of 0.24 mg/mL, a *V*_max_ value of 4.156 μM/min·mg, a *k_cat_* value of 41.56 s^−1^, and a *k_cat_*/*K*_m_ of 175.73 mL/mg·s. Notably, when colloidal chitin is used as a substrate, *Cm*Chi exhibits a *K*_m_ value that is lower than that of most other reported chitinases (Table 2), indicating a high substrate affinity. More importantly, the *k_cat_*/*K*_m_ of *Cm*Chi, 175.73 mL/mg·s, is higher than that of most chitinases (Table 2), underscoring its potential for efficient chitin hydrolysis. These findings demonstrate that *Cm*Chi possesses both high substrate affinity and remarkable catalytic efficiency for colloidal chitin.

**Table 2:** Michaelis constant (*K*_m_) and catalytic efficiency (*k_cat_*/*K*_m_) of various chitinases with colloidal chitin substrate.

Enzyme	Microbial source	*K*_m_ (mg/mL)	*k_cat_*/*K*_m_ (mL/mg·s)	Ref
*Cm*Chi	*C. mangrovi* FCG-7^T^	0.24	175.73	This study
*Pb*Chi67	*P. barengoltzii*	3.35	0.0056	Fu et al. (2016)
ChiA-Hh59	*H. hirschii* strain KB-DZ44	0.30	48,322	Bouacem et al. (2018)
Chitinase	*A. griseoaurantiacus* KX010988	0.22	0.69	Shehata et al. (2018)
Chi1	*S. thermodiastaticus* HF 3-3	1.23 ± 0.70	—	Take et al. (2018)
*Cm*Chi3	*C. meiyuanensis*	7.53 ± 0.78	1.2 ± 0.11	Wang et al. (2022)
ChiC8-1	*Chitinilyticum* sp. C8	10.17	—	Zhao et al. (2023)
chitinase	*B. aryabhattai*	4.60	16	Subramani et al. (2024)
*Td*ChiT	*T. dupontii*	0.34	74.17	Kumari et al. (2024)
*Ca*Chi18A	*C. aquatile* CSC-1	0.94	26.34	Chen et al. (2024a, b)
ChiA74	*B. thuringiensis*	32.65 ± 8.47	1.215	Martínez-Zavala et al. (2025)
Chi1	*Chitinibacter* sp. GC72.	0.76 ± 0.03	16.435 ± 0.87	Zhang et al. (2025)
Chit33	*Trichoderma harzianum*	4.60 ± 0.70	1.3 ± 0.2	Martínez-Ranz et al. (2025)
*Cg*chi18	*Chaetomium globosum* W7	3.18	—	Jiang et al. (2025)
*Mp*Chit35	*Metschnikowia pulcherrima*	25.00 ± 5.00	0.06 ± 0.01	Minguet-Lobato et al. (2025)
MBJ7879808.1	*Gelidibacter salicanalis* PAMC21136	2.86	2.0	Paudel et al. (2025)

### 3.8 Bifunctional hydrolysis properties of *Cm*Chi

**Figure 5:**
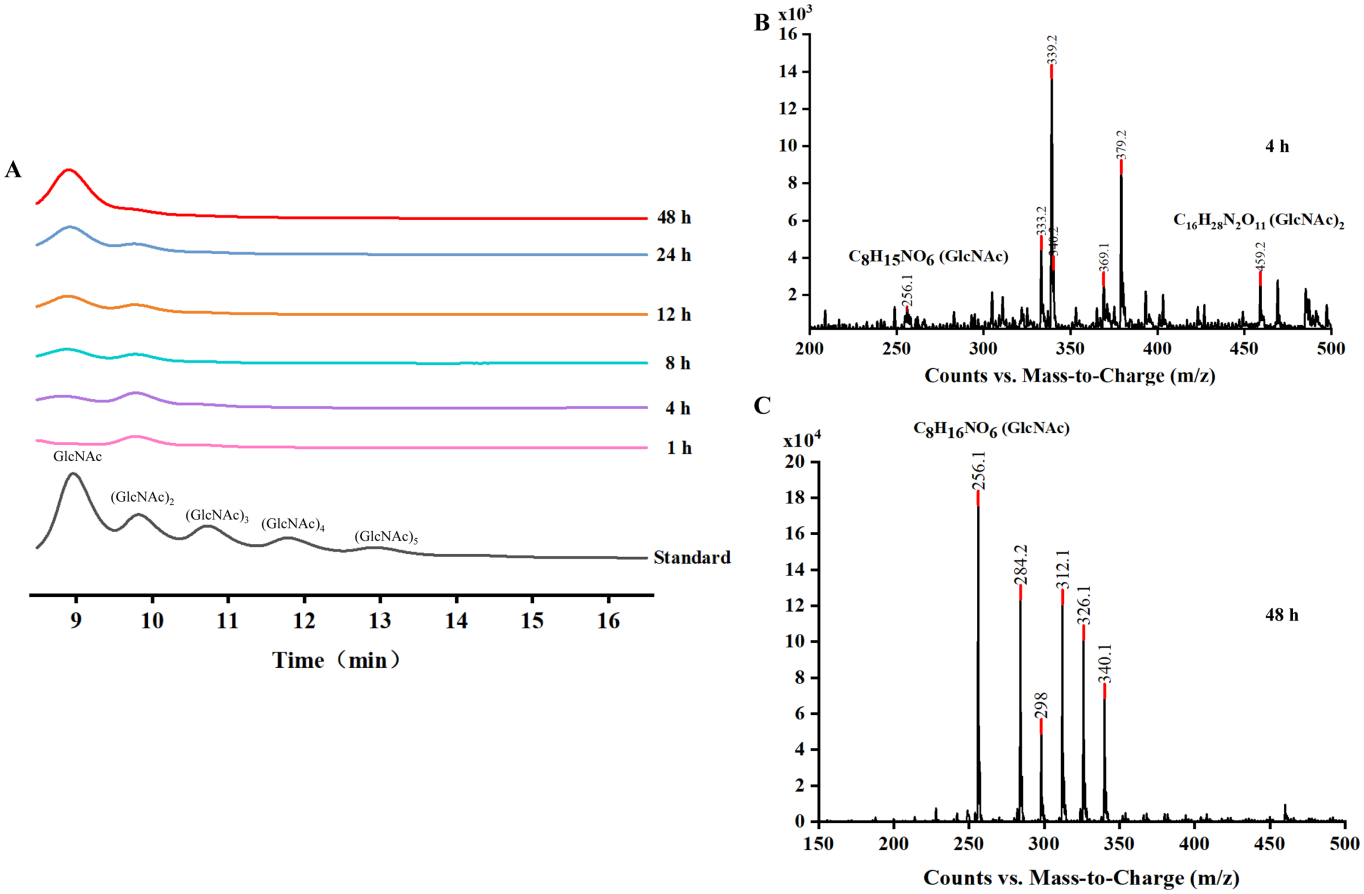
Analysis of hydrolysis products. **(A)** HPLC analysis of hydrolysates from colloidal chitin digested with *Cm*Chi. The standard is chitin oligosaccharides (degree of polymerization 1–6, DP). **(B)** LC–MS/MS analysis of hydrolysates from colloidal chitin digested with *Cm*Chi at 4 h. **(C)** LC–MS/MS analysis of hydrolysates from colloidal chitin digested with *Cm*Chi at 48 h.

HPLC analysis demonstrated that (GlcNAc)_2_ was the predominant product in the early stages of hydrolysis, indicating chitobiosidase activity of *Cm*Chi. As the reaction progressed, (GlcNAc)_2_ was gradually converted into GlcNAc. By 48 h, GlcNAc became the primary hydrolysis product (Figure 5A), confirming that *Cm*Chi hydrolyzes (GlcNAc)_2_ into GlcNAc and thus exhibits NAGase activity. LC–MS/MS analysis, performed in negative ion mode, further supported these findings. At the 4-h time point (Figure 5B), the mass spectrum displayed prominent signals at m/z 256.1 and 459.2, which correspond to the chloride adducts [M + Cl]^−^ of GlcNAc (theoretical m/z 256.21) and (GlcNAc)₂ (theoretical m/z 459.40), respectively. Chloride adducts are commonly observed in negative-ion ESI-MS analysis of neutral carbohydrates, particularly when chloride ions are present in the mobile phase or sample matrix (Wan and Yu, 2007; Vinueza et al., 2013). Several additional ion signals (e.g., m/z 339.2, 379.2, 419.2, 498.3) were also present; however, their m/z values did not match the adducts of any known chitin oligosaccharides or their common derivatives, and they were therefore not further assigned. By 48 h (Figure 5C), the (GlcNAc)_2_-related peak at m/z 459.2 had substantially diminished, while the GlcNAc [M + Cl]^−^ peak at m/z 256.1 remained predominant, confirming the nearly complete conversion of chitin to GlcNAc and the inherent NAGase activity of *Cm*Chi. To quantitatively verify the NAGase activity of *Cm*Chi toward (GlcNAc)_2_, a fluorometric assay was performed using 4-MU-GlcNAc as substrate. *Cm*Chi exhibited an activity of 2.09 U/mg, providing independent confirmation of its inherent NAGase activity. This finding is consistent with the HPLC and LC–MS/MS results described above.

Together, these results establish *Cm*Chi as a bifunctional chitinase with both chitobiosidase and NAGase activities. After 48 h of hydrolysis at 30 °C, the end products were almost exclusively GlcNAc. In contrast to monofunctional chitinases, *Cm*Chi’s dual activity prevents the accumulation of oligomeric intermediates such as (GlcNAc)_2_. By efficiently hydrolyzing both long-chain chitin and (GlcNAc)_2_, *Cm*Chi enhances the purity and yield of GlcNAc, underscoring its promising potential for applications in green biomanufacturing.

### 3.9 Immunomodulatory effects of GlcNAc in CTX-treated mice

#### 3.9.1 Effects of GlcNAc on body weight and immune organ indices in CTX-treated mice

**Figure 6:**
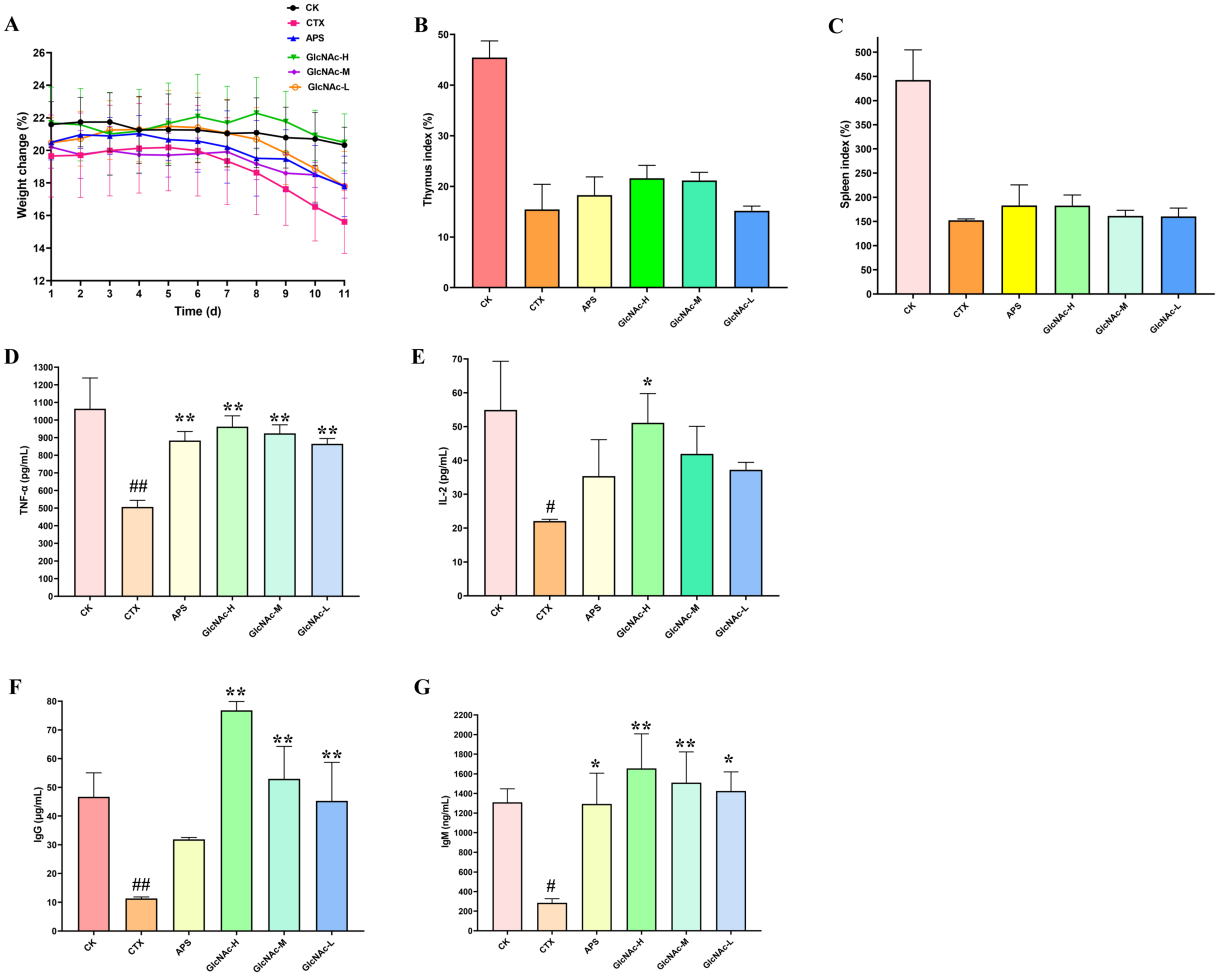
Effect of *N*-acetylglucosamine (GlcNAc) on body weight, immune organ indices, and serum levels of cytokines and immunoglobulins in cyclophosphamide (CTX)-induced immunosuppressed mice. **(A)** Daily body weight changes. **(B)** Thymus index. **(C)** Spleen index. **(D)** Serum tumor necrosis factor-alpha (TNF-*α*) levels. **(E)** Serum interleukin-2 (IL-2) levels. **(F)** Serum immunoglobulin G (IgG) levels. **(G)** Serum immunoglobulin M (IgM) levels. CK, normal control (saline); CTX, model group (80 mg/kg/day CTX); APS, positive control (100 mg/kg/day Astragalus polysaccharide); GlcNAc-H, high-dose GlcNAc (200 mg/kg/day); GlcNAc-M, medium-dose GlcNAc (100 mg/kg/day); GlcNAc-L, low-dose GlcNAc (50 mg/kg/day). Data are presented as mean ± SE from three independent replicates (*n* = 3). Hash symbols indicate statistically significant differences compared with the CK group (Student’s *t*-test): #*p* < 0.05, ##*p* < 0.01. Asterisks indicate statistically significant differences compared with the CTX group (Student’s *t*-test): *p < 0.05, **p < 0.01.

Body weight is a key indicator of overall health status, with its dynamic changes directly reflecting physiological homeostasis. As shown in Figure 6A, all groups maintained stable body mass during the initial 6-day period. After modeling induction on day 7, differential weight reduction was observed across groups, confirming successful establishment of the CTX-induced immunosuppressive model. The slight decline in the CK group may be attributed to stress responses from intraperitoneal injection. Notably, compared to the CTX group, the APS, GlcNAc-H, and GlcNAc-M groups showed significant mitigation of CTX-induced weight loss. In contrast, the GlcNAc-L group exhibited no significant ameliorative effect.

The thymus and spleen are central immune organs critical for immunological regulation, and their functional status directly influences immune competence (Cai et al., 2021). Immune organ indices serve as reliable markers of physiological health, with decreased values indicating atrophy or degenerative changes-well-established signs of immunosuppression (Ding et al., 2019; Xie et al., 2022). As illustrated in Figures 6B,C, CTX administration led to significant reductions in both thymic and splenic indices compared to the CK group (*p* < 0.01), further validating the immunosuppressive model. Moreover, both APS and GlcNAc-H/M treatments significantly alleviated CTX-induced damage to immune organs relative to CK controls (*p* < 0.05).

To further confirm the effect of GlcNAc on thymus and spleen tissue structures in CTX-treated mice, pathological sections and changes were observed (Supplementary Figure 5). CK thymi showed sharp cortico-medullary borders, intact Hassall’s corpuscles and dense lymphocytes (Supplementary Figure 5B). CTX erased corpuscles, blurred borders and thinned the cortex. APS partially rescued lymphocyte density but borders remained indistinct. GlcNAc restored architecture dose-dependently: the 200 mg/kg group re-established clear cortico-medullary differentiation and normal corpuscle outlines. CK spleens exhibited well-demarcated white and red pulp with abundant lymphoid follicles. CTX shrank white pulp, effaced boundaries and increased reticular/fibroblast infiltrates. APS gave only modest improvement. High-dose GlcNAc again produced the strongest repair-white pulp expanded, follicles became visible and lymphocyte counts rose (Supplementary Figure 5B). Collectively, GlcNAc reverses CTX-induced immune-organ atrophy in a dose-responsive manner.

#### 3.9.2 Effects of GlcNAc on cytokines immunoglobulin secretion level in CTX-treated mice

Cytokines, primarily secreted by activated immune cells, play diverse roles in immune regulation. For instance, IL-2 is involved in both Th1 and Th2 immune responses (Tang et al., 2018), while IgG secretion reflects humoral immune function (Flint et al., 2015). As shown in Figures 6D–G, mice treated with CTX exhibited significantly reduced serum levels of TNF-*α*, IL-2, IgG, and IgM compared to the CK group (*p* < 0.05). Treatment with APS and GlcNAc markedly restored these cytokine and immunoglobulin levels. Moreover, GlcNAc exerted a dose-dependent restorative effect (*p* < 0.05), with the high-dose group showing significantly greater efficacy than the APS group in elevating TNF-α, IL-2, IgG, and IgM (*p* < 0.05). Although Cheng et al. (2014) previously demonstrated the immunomodulatory activity of GlcNAc in tilapia, this study is the first to confirm its efficacy in a CTX-induced immunosuppressed mouse model, showing that 200 mg/kg GlcNAc significantly elevates serum TNF-α, IL-2, IgG, and IgM.

## 4 Discussion

This study successfully isolated and characterized a novel chitinolytic bacterium, *C. mangrovi* FCG-7^T^, from mangrove sediments. The strain demonstrated exceptional chitin degradation capacity with an H/C ratio of 4.75, which significantly exceeds the values reported for related strains: *Chitinibacter* sp. SCUT-21 (4.66) (Yang et al., 2023), *Chitinilyticum* sp. C8 (3.7) (Zhao et al., 2025), *B. salmalaya* 139SI (3.0) (Alsalman et al., 2022), and *Micromonospora* sp. ATLC16 (2.6) (Chaulagain et al., 2024), and maintained genetic stability over ten generations, underscoring its industrial potential.

Through a systematic optimization strategy employing OFAT, PBD, and BBD, chitinase production was enhanced by 49.43-fold, achieving an activity of 1.211 U/mL. PBD effectively screened for significant factors, identifying initial pH, chitin powder concentration, and temperature as the primary drivers of enzyme yield, a finding consistent with previous studies on chitinase optimization (Lee and Kim, 2015; Rishad et al., 2016; Pasqualetti et al., 2022; Xie et al., 2025). The high significance of the quadratic model (*p* < 0.0001) from the subsequent BBD analysis confirmed strong interactions between the variables, and the close alignment of the predicted and validated results confirms the robustness of the model. To the best of our knowledge, this constitutes the first report on chitinase production and process optimization in *C. mangrovi* FCG-7^T^.

The purified chitinase, *Cm*Chi, is a major 100 kDa bifunctional enzyme with both chitobiosidase and NAGase activities. While its molecular weight is larger than many typical bacterial chitinases (20–80 kDa) (Sakai et al., 1998; Mander et al., 2016; Bouacem et al., 2018; Fu et al., 2016). In addition to the 100 kDa band, a faint but reproducible activity band at approximately 70 kDa was consistently observed in the zymogram (Figure 3C, lane 2 and 3). Genome analysis of *C. mangrovi* FCG-7^T^ revealed the presence of multiple chitinase-encoding genes (Xu et al., 2025), suggesting that this ~70 kDa band likely represents a distinct, smaller chitinase isoenzyme constitutively expressed under the tested fermentation conditions. The presence of this minor isoenzyme indicates the co-expression of multiple chitinases, a phenomenon also observed in other chitinolytic bacteria such as *Chitinolyticbacter meiyuanensis* (Zhang et al., 2020) and *Chitiniphilus shinanonensis* (Okazaki et al., 2023). In the present study, we focused on the major 100 kDa *Cm*Chi due to its dominant activity, high specific activity (7.96 U/mg), and successful purification to apparent homogeneity. Characterization of the ~70 kDa isoenzyme will be pursued in future investigations.

*Cm*Chi exhibited optimal activity at 50 °C and pH 6.0, which is comparable to many mesophilic bacterial chitinases (Aktas et al., 2023; Xie et al., 2025; Dikbaş et al., 2023; Sharma et al., 2020). Moreover, *Cm*Chi displays favorable thermostability up to 45 °C. The thermal stability profile is comparable to that of recombinant CHI (Liu et al., 2020) and exceeds the thermostability of several chitinases, including *Ca*Chi18A (Chen et al., 2024a), *Ca*Chi18B (Gu et al., 2025), and chitinase from *Vibrio maritimus* (Dikbaş et al., 2025), which retain stability only up to 40 °C. The thermostability of *Cm*Chi is likely governed by its intrinsic structural features, such as compact folding, enhanced hydrophobic interactions, and an increased number of salt bridges and hydrogen bonds, which are known to stabilize protein conformation at elevated temperatures (Malecki et al., 2020; Dou et al., 2024; Li et al., 2025). Environmental factors, including the fluctuating intertidal conditions of the native mangrove ecosystem, may have exerted evolutionary pressure that favored the selection of such thermostable structural traits (Marasco et al., 2023). The enzyme also displayed remarkable pH stability across a broad range (pH 4.0–11.0). This wide pH tolerance surpasses that of many other characterized chitinases (Zhao et al., 2023; Wu et al., 2022; Zhang et al., 2018; Fu et al., 2020). The significant inhibition by Cu^2+^ is a common feature of many chitinases (Wang et al., 2010; Lee et al., 2018; Du et al., 2021), and is likely due to coordination with active site cysteine residues, leading to conformational changes (Wang et al., 2010; He et al., 2022). The enzyme’s tolerance to several organic solvents further highlights its robustness for industrial application.

*Cm*Chi displayed the highest activity toward colloidal chitin, followed by *β*-chitin and *α*-chitin. This pattern aligns with the general characteristics reported for most chitinases (Liu et al., 2024; Yu et al., 2024). The pronounced hydrolytic capability of *Cm*Chi toward colloidal chitin contrasts with its weaker activity toward α-chitin, a difference may be attributed to variations in crystallinity. α-chitin possesses a highly crystalline structure due to its antiparallel chain arrangement and extensive hydrogen bonding, which hinders enzyme penetration and action, whereas colloidal chitin is amorphous and more accessible to enzymatic attack (Cardozo et al., 2019; Hou et al., 2021; Chen et al., 2024b).

Kinetic analysis revealed that *Cm*Chi has a high substrate affinity for colloidal chitin, with a *K*_m_ of 0.24 mg/mL, which is lower than most reported chitinases (Table 2). More importantly, its catalytic efficiency (*k_cat_*/*K*_m_) of 175.73 mL/mg·s is superior to several well-studied enzymes like TdChiT (74.17 mL/mg·s; Kumari et al., 2024) and CaChi18A (26.34 mL/mg·s; Chen et al., 2024a) (Table 2). This high efficiency is functionally validated by its complete conversion of colloidal chitin to GlcNAc within 48 h. The dual chitobiosidase and NAGase activities of *Cm*Chi, confirmed by HPLC and LC–MS/MS, are particularly noteworthy. This bifunctionality allows for the direct production of monomeric GlcNAc without the accumulation of oligosaccharide intermediates such as (GlcNAc)_2_, a common bottleneck in enzymatic chitin degradation that complicates downstream purification (Fu et al., 2014; Zhang et al., 2018). This feature distinguishes *Cm*Chi as a highly promising biocatalyst for the efficient and green production of GlcNAc.

Beyond direct GlcNAc production, the excellent biochemical properties of *Cm*Chi, including its broad pH stability, high substrate affinity, and tolerance to organic solvents, make it an ideal candidate for further engineering and process integration. For instance, immobilization of chitinase onto magnetic nanoparticles has been shown to enhance thermal stability and enable easy enzyme recovery, as demonstrated for VhChiA from *Vibrio harveyi*, which retained nearly 40% activity after 16 reaction cycles (Charoenpol et al., 2024). Such an approach could be applied to *Cm*Chi to facilitate continuous bioprocessing. Furthermore, a three-step enzymatic strategy employing engineered chitinase (TfChit mutants), hypertransglycosylating β-N-acetylhexosaminidase, and peptidoglycan deacetylase has been successfully developed to convert chitin into partially deacetylated chitooligomers with degree of polymerization 6–11 (Mészáros et al., 2024). Given that *Cm*Chi efficiently produces GlcNAc and (GlcNAc)_2_, it could serve as a starting module in similar cascade reactions to generate tailored, bioactive chitooligosaccharides for agricultural or pharmaceutical applications.

Finally, this study provides the first evidence of GlcNAc’s immunomodulatory effects in a mammalian CTX-induced immunosuppression model. The restoration of body weight, immune organ indices, and tissue architecture, along with the dose-dependent elevation of serum TNF-*α*, IL-2, IgG, and IgM, clearly demonstrates GlcNAc’s efficacy in reversing chemotherapy-induced immunosuppression. The observed effects likely stem from GlcNAc’s role in stabilizing key immune signaling pathways. As a key substrate for O-GlcNAc modification, exogenous GlcNAc can enhance protein O-GlcNAcylation via the hexosamine biosynthesis pathway (Vocadlo et al., 2003; Yang and Qian, 2017). This post-translational modification is crucial for lymphocyte activation and the regulation of transcription factors such as NF-κB and NFAT, thereby promoting cytokine production (Qiang et al., 2021). Furthermore, the recovery of IgG and IgM levels suggests GlcNAc supports B-cell function and humoral immunity, processes that depend on proper antibody glycosylation (Paneque et al., 2023). The results align with and significantly extend previous findings on the immunomodulatory roles of GlcNAc in other contexts: GlcNAc has been shown to activate the NRF2/HO-1 pathway and modulate macrophage polarization (Xu et al., 2023), and to exert immunomodulatory effects in tilapia (Cheng et al., 2014). Together, these findings provide a strong foundation for the potential development of GlcNAc as a supportive immunotherapeutic agent.

## 5 Conclusion

This study establishes *C. mangrovi* FCG-7^T^ as a robust and genetically stable bacterium with high chitin degradation efficiency. Through systematic fermentation optimization, chitinase production was significantly enhanced by 49.43-fold, culminating in the purification of a bifunctional enzyme, *Cm*Chi. This enzyme efficiently hydrolyzes chitin to GlcNAc within 48 h. *Cm*Chi exhibits remarkable biochemical properties, including high affinity for colloidal chitin (*K_m_* = 0.24 mg/mL), broad pH stability (pH 4.0–11.0), and solvent tolerance, underscoring its suitability for industrial GlcNAc production. Moreover, GlcNAc administration (200 mg/kg) significantly alleviated CTX-induced immunosuppression in mice, restoring immune organ indices, cytokine levels (TNF-*α*, IL-2), and immunoglobulin profiles (IgG, IgM). To our knowledge, this is the first report to validate the immunomodulatory efficacy of GlcNAc in a mammalian CTX-induced immunosuppression model. These findings position *Cm*Chi as a sustainable biocatalyst for chitin valorization, while GlcNAc emerges as a promising immunotherapeutic agent. Looking forward, heterologous expression of *Cm*Chi and protein engineering will be pursued to enhance production scalability, while transcriptomic and pharmacological studies will be conducted to elucidate the molecular mechanisms underlying GlcNAc-mediated immune restoration and to advance its translational potential.

## Data Availability

The original contributions presented in the study are included in the article/Supplementary material, further inquiries can be directed to the corresponding authors.
