# Supplementary material for: Highly efficient chitinase production from Chitinibacter mangrovi FCG-7T and immunomodulatory efficacy of generated GlcNAc in cyclophosphamide-induced immunosuppression
Source: Front Microbiol. 2026 May 11;17:1826206. doi: 10.3389/fmicb.2026.1826206 (PMC13199048; doi:10.3389/fmicb.2026.1826206)
Supplement: Supplementary file 1 [file Supplementary_file_1.docx]

Supplementary Material


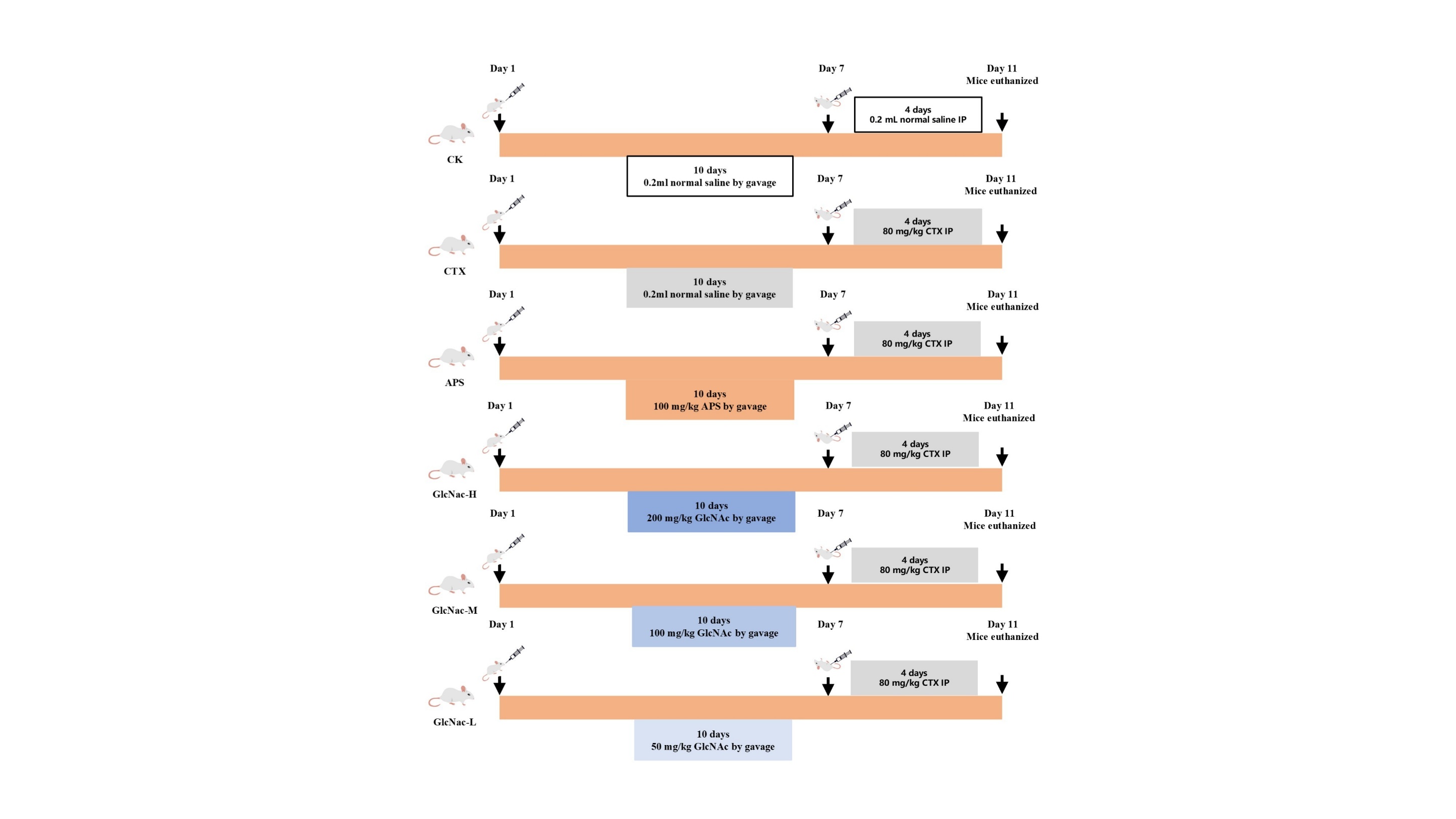


**Supplementary Figure 1.** Animal experiment protocol.


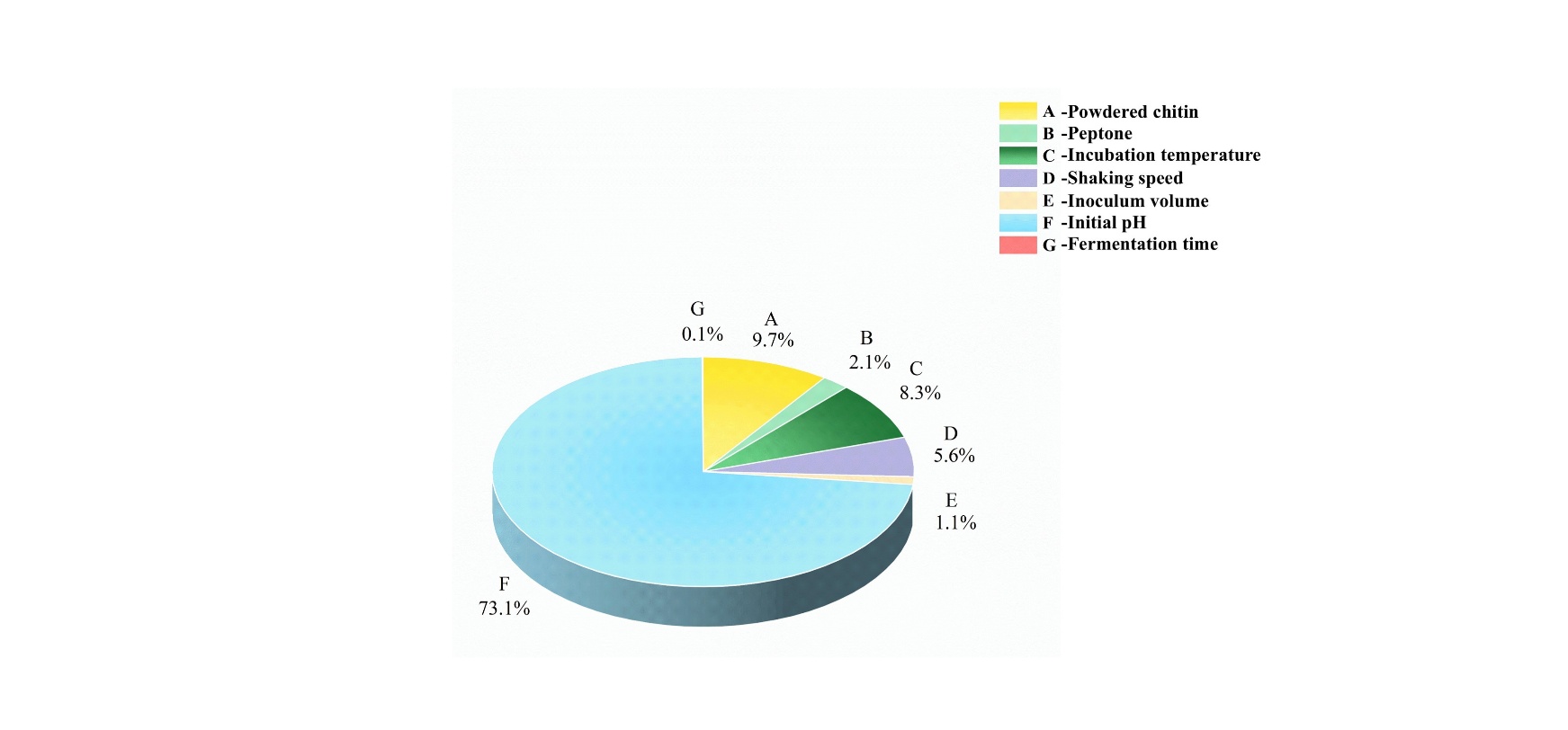


**Supplementary Figure 2.** An exploration of the impact of individual components on chitinase production by *C. mangrovi* FCG-7^T^ utilizing a Plackett-Burman model.


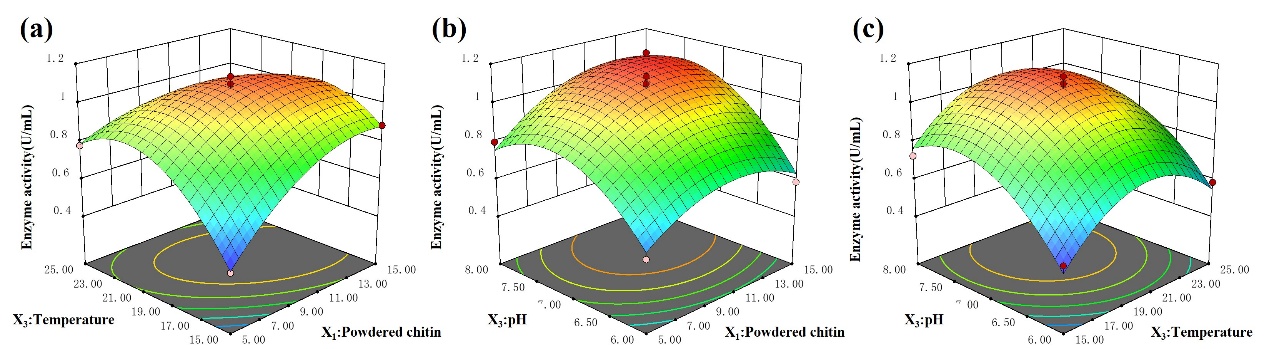


**Supplementary Figure 3.** Response surface analysis diagram of the interaction of various factors on enzyme activity. (a). Effects of temperature and powdered chitin addition on enzyme activity (b). Effects of pH and powdered chitin addition on enzyme activity. (c). Effects of pH and temperature on enzyme activity.





**Supplementary Figure 4.** Elution profile of DEAE-52 column. P1-P5: Eluted protein fractions.


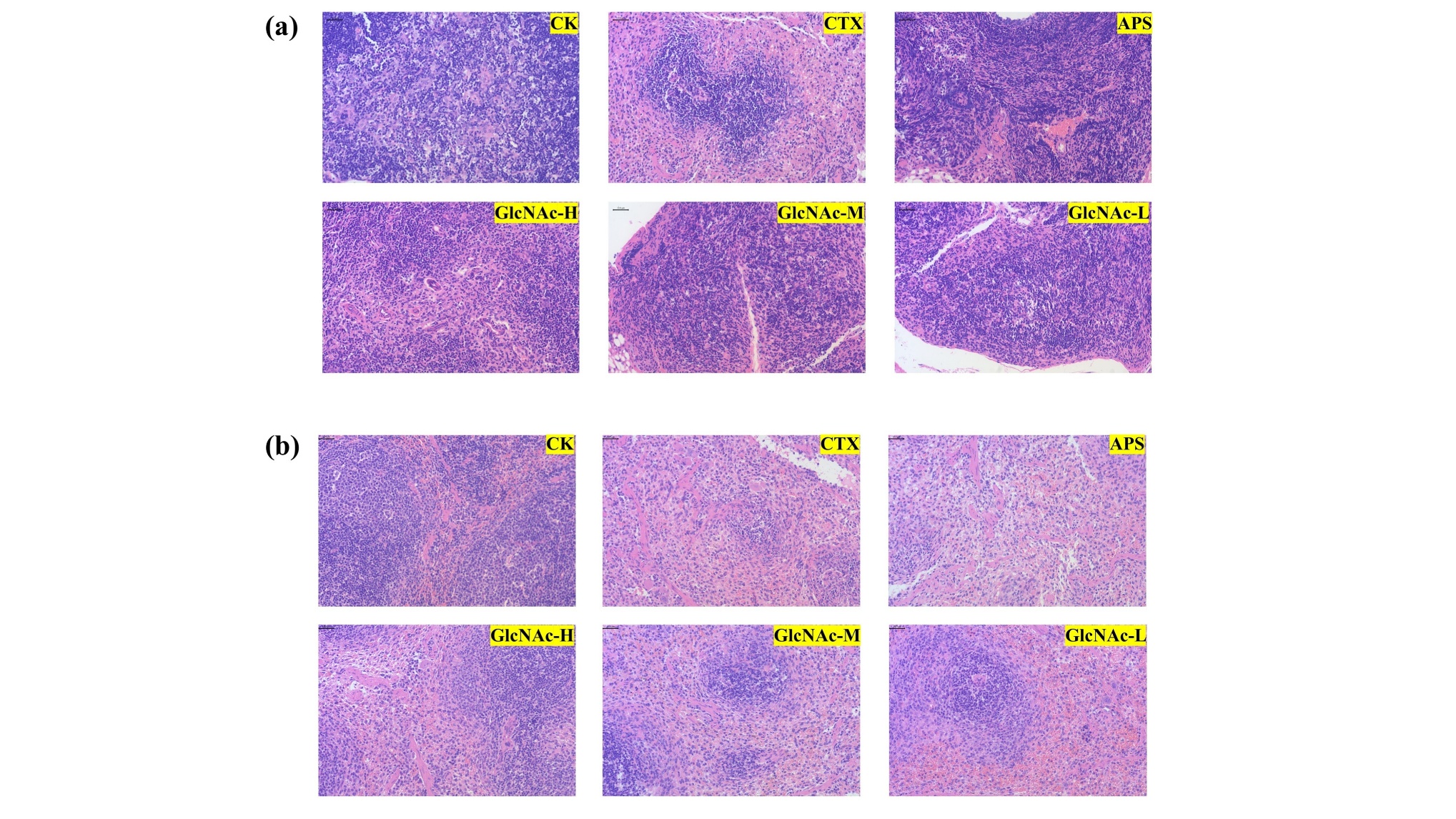


**Supplementary Figure 5.** Effects of GlcNAc on thymus and spleen tissue structures in CTX-treated mice. (a) Microscopic analysis of thymus sections (HE200×). (b) Microscopic analysis of Spleen sections (HE200×).

**Table S1.** Factors and variables of one-factor-at-a-time optimization.

| Factors | Variables |
| --- | --- |
| Carbon source | Powdered chitin, Colloidal chitin, Lactose, Glucose, Sucrose |
| Nitrogen source | Peptone, Yeast extract, Beef paste, Urea, Ammonium sulfate |
| Powdered chitin (g/L) | 2.5，5，10，20，30 |
| Peptone (g/L) | 2.5，5，10，20，30 |
| Initial pH | 7，8，9，10，11 |
| Temperature (℃) | 20，25，30，35，40 |
| Shaking speed (r/min) | 180，200，220，240，260 |
| Inoculum volume (%) | 2，3，4，5，6 |
| Fermentation time (h) | 24，48，72，96，120，144，168 |

**Table S2.** PBD factor and levels coding values

| Factor code | Factor | Level | |
| --- | --- | --- | --- |
|  |  | Low (-1) | High (+1） |
| A | Powdered chitin (g/L) | 5 | 15 |
| B | Peptone (g/L) | 5 | 15 |
| C | Temperature (℃) | 15 | 25 |
| D | Shaking speed (r/min) | 220 | 260 |
| E | Inoculum volume (%) | 3 | 5 |
| F | Initial pH | 6 | 8 |
| G | Fermentation time (h) | 72 | 120 |

**Table S3.** Test factors and level coding values

| Factor | Level | | |
| --- | --- | --- | --- |
|  | -1 | 0 | 1 |
| X_1_-Powdered chitin (g/L) | 5 | 10 | 15 |
| X_2_-Temperature (℃) | 15 | 20 | 25 |
| X_3_-Initial pH | 6 | 7 | 8 |

**Table S4.** Plackett-Burman experimental results

| No. | A | B | C | D | E | F | G | Enzyme activity  (U/mL) |
| --- | --- | --- | --- | --- | --- | --- | --- | --- |
| 1 | -1 | -1 | 1 | -1 | 1 | 1 | -1 | 0.467 |
| 2 | 1 | 1 | -1 | -1 | -1 | 1 | -1 | 0.548 |
| 3 | 1 | -1 | -1 | -1 | 1 | -1 | 1 | 0.013 |
| 4 | -1 | 1 | 1 | 1 | -1 | -1 | -1 | 0.216 |
| 5 | -1 | -1 | -1 | -1 | -1 | -1 | -1 | 0.003 |
| 6 | 1 | 1 | -1 | 1 | 1 | 1 | -1 | 0.590 |
| 7 | -1 | 1 | 1 | -1 | 1 | 1 | 1 | 0.439 |
| 8 | -1 | 1 | -1 | 1 | 1 | -1 | 1 | 0.149 |
| 9 | 1 | -1 | 1 | 1 | -1 | 1 | 1 | 0.674 |
| 10 | -1 | -1 | -1 | 1 | -1 | 1 | 1 | 0.451 |
| 11 | 1 | 1 | 1 | -1 | -1 | -1 | 1 | 0.349 |
| 12 | 1 | -1 | 1 | 1 | 1 | -1 | -1 | 0.324 |

**Table S5.** ANOVA for the PBD

| Source | df | Sum of Squares | Mean Square | F-value | *p*-value | Significance |
| --- | --- | --- | --- | --- | --- | --- |
| Model | 7 | 0.5104 | 0.0729 | 18 | 0.0071 | ** |
| A-Powdered chitin | 1 | 0.0497 | 0.0497 | 12.28 | 0.0248 | * |
| B-Peptone | 1 | 0.0108 | 0.0108 | 2.67 | 0.1774 |  |
| C-Incubation temperature | 1 | 0.0423 | 0.0423 | 10.45 | 0.0319 | * |
| D-Shaking speed | 1 | 0.0284 | 0.0284 | 7.01 | 0.0571 |  |
| E-Inoculum volume | 1 | 0.0055 | 0.0055 | 1.37 | 0.3073 |  |
| F-Initial pH | 1 | 0.3732 | 0.3732 | 92.11 | 0.0007 | ** |
| G-Fermentation time | 1 | 0.0004 | 0.0004 | 0.1057 | 0.7613 |  |
| Residual | 4 | 0.0162 | 0.0041 |  |  |  |
| Cor Total | 11 | 0.5266 |  |  |  |  |
| R^2^ | 0.9692 |  |  | Pred R^2^ | 0.7231 |  |
| R^2^_adj_ | 0.9154 |  |  | Adeq Precisior | 13.1923 |  |

** Indicates extremely significant difference, * indicates significant difference

**Table S6.** BBD experimental results.

| No. | X_1_ | X_2_ | X_3_ | Enzyme activity(U/mL) |
| --- | --- | --- | --- | --- |
| 1 | 0 | 0 | 0 | 1.065 |
| 2 | 0 | 0 | 0 | 1.099 |
| 3 | -1 | -1 | 0 | 0.438 |
| 4 | 0 | 1 | 1 | 0.793 |
| 5 | 0 | -1 | -1 | 0.474 |
| 6 | 0 | 1 | -1 | 0.584 |
| 7 | 1 | -1 | 0 | 0.886 |
| 8 | 0 | -1 | 1 | 0.725 |
| 9 | 1 | 1 | 0 | 0.725 |
| 10 | 1 | 0 | -1 | 0.586 |
| 11 | -1 | 0 | -1 | 0.504 |
| 12 | 1 | 0 | 1 | 1.064 |
| 13 | -1 | 0 | 1 | 0.799 |
| 14 | 0 | 0 | 0 | 1.034 |
| 15 | 0 | 0 | 0 | 1.136 |
| 16 | -1 | 1 | 0 | 0.780 |
| 17 | 0 | 0 | 0 | 1.052 |

**Table S7.** BBD experimental results.

| Source | Sum of Squares | df | Mean Square | F-value | p-value |  |
| --- | --- | --- | --- | --- | --- | --- |
| Model | 0.8769 | 9 | 0.0974 | 35.71 | < 0.0001 | significant |
| X_1_ | 0.0683 | 1 | 0.0683 | 25.04 | 0.0016 |  |
| X_2_ | 0.0162 | 1 | 0.0162 | 5.92 | 0.0452 |  |
| X_3_ | 0.1902 | 1 | 0.1902 | 69.7 | < 0.0001 |  |
| X_1_X_2_ | 0.0632 | 1 | 0.0632 | 23.18 | 0.0019 |  |
| X_1_X_3_ | 0.0083 | 1 | 0.0083 | 3.04 | 0.1247 |  |
| X_2_X_3_ | 0.0005 | 1 | 0.0005 | 0.171 | 0.6916 |  |
| X_1_^2^ | 0.08 | 1 | 0.08 | 29.33 | 0.001 |  |
| X_2_^2^ | 0.2266 | 1 | 0.2266 | 83.05 | < 0.0001 |  |
| X_3_^2^ | 0.1707 | 1 | 0.1707 | 62.56 | < 0.0001 |  |
| Residual | 0.0191 | 7 | 0.0027 |  |  |  |
| Lack of Fit | 0.0125 | 3 | 0.0042 | 2.51 | 0.1973 | Not significant |
| Pure Error | 0.0066 | 4 | 0.0017 |  |  |  |
| Cor Total | 0.896 | 16 |  |  |  |  |

**Table S8.** Purification summary of chitinase from *C. mangrovi* FCG-7^T^

| Purification steps | Total protein (mg) | Total chitinase activity (U) | Specific activity (U/mg) | Purification fold | Yield (%) |
| --- | --- | --- | --- | --- | --- |
| Crude enzyme | 1123 | 122.30 | 0.11 | 1 | 100 |
| Ammonium sulfate | 43.8 | 24.97 | 0.57 | 5.18 | 20.42 |
| DEAE-52 | 2.53 | 20.14 | 7.96 | 72.36 | 16.47 |
